# Preanalytical, analytical and postanalytical considerations in circulating microRNAs measurement

**DOI:** 10.11613/BM.2024.020501

**Published:** 2024-06-15

**Authors:** Mustapha Zendjabil

**Affiliations:** 1 Faculty of Medicine, University of Oran 1 - Ahmed Ben Bella, Oran, Algeria; 2 Department of Biochemistry, Oran University Hospital, Oran, Algeria

**Keywords:** microRNAs, preanalytical phase, blood collection, biomarkers, liquid biopsy

## Abstract

Microribonucleic acids (miRNAs) have emerged as a new category of biomarkers for many human diseases like cancer, cardiovascular and neurodegenerative disorders. MicroRNAs can be detected in various body fluids including blood, urine and cerebrospinal fluid. However, the literature contains conflicting results for circulating miRNAs, which is the main barrier to using miRNAs as non-invasive biomarkers. This variability in results is largely due to differences between studies in sample processing methodology, miRNA quantification and result normalization. The purpose of this review is to describe the various preanalytical, analytical and postanalytical factors that can impact miRNA detection accuracy and to propose recommendations for the standardization of circulating miRNAs measurement.

## Graphical abstract

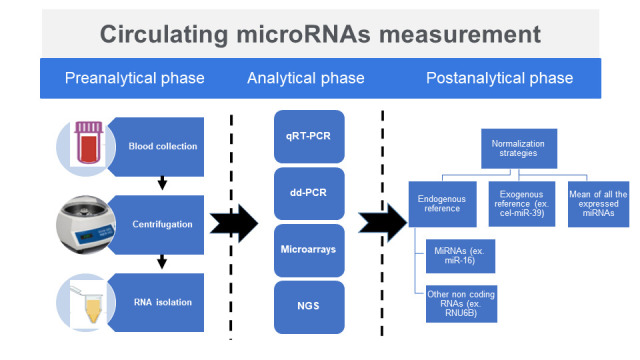

## Highlights

• Methods used for miRNAs expression profiling are quantitative reverse transcription polymerase chain reaction, microarrays, next generation sequencing and droplet digital PCR

• To obtain reproducible and accurate miRNAs expression profiling detection, it is crucial to strictly standardize the entire process, starting from choosing the specimen type until the normalization strategy on the interpretation of miRNAs expression profiling detection

•Due to the critical impact of the normalization strategy on the miRNAs expression, the choice of normalization agent is of great importance

## Introduction

Microribonucleic acids (miRNAs), consisting of approximately 20 nucleotides, belong to a non-coding class of ribonucleic acids. They play a crucial role in regulating numerous biological processes at the posttranscriptional level by binding to their corresponding messenger ribonucleic acids (mRNAs). This interaction ultimately results in either the degradation or inhibition of mRNA translation (*1*). Thanks to their different forms of transport, miRNAs are stable and can be detected in biological fluids such as blood, urine, cerebrospinal fluid and saliva. The expression of miRNAs varies significantly in quality and quantity across different blood compartments. Microribonucleic acids are particularly abundant in exosomes, which are essential mediators of intercellular communication (*2*). Moreover, the profile of circulating miRNAs is partly specific to the cells that secreted them and is modified according to the physiological or pathological cellular state (*3*). Microribonucleic acids are currently emerging as promising biomarkers for various human diseases. The first study to have demonstrated the interest of miRNAs as biomarkers is that of Lawrie *et al.* in 2008 (*4*). Since then, many miRNAs signatures have been described mainly for cancerous diseases, but also for other human pathologies such as cardiovascular and neurodegenerative diseases (*5*). However, we note in literature that the results of circulating miRNAs are often contradictory. This inconsistency poses a major challenge to the potential utilization of miRNAs as non-invasive biomarkers and raises doubts about the clinical validity of miRNAs expression in peripheral blood. The aim of this review is to describe different preanalytical, analytical and postanalytical variables that can influence the measurement of circulating miRNAs and to propose recommendations for the standardization of circulating miRNAs measurement.

## Preanalytical phase

A well-regulated preanalytical phase within the laboratory is crucial for maintaining the accuracy and reliability of test results. In fact, it is widely acknowledged that a significant majority, approximately 60 to 70%, of errors encountered in the laboratory arise during this particular phase (*6*).

### Samples

It is important to acknowledge that sample collection timing can influence the miRNA analysis outcomes. This is due to the fact that certain miRNAs exhibit rhythmic variations in their concentrations, depending on time of the day when the specimen was obtained (*7*). Microribonucleic acids can be extracted from a variety of specimen types: cells in culture, fresh or fixed tissue/tumors or biological fluids such as serum, plasma, saliva, urine and cerebrospinal fluid (*8*). Even if miRNAs are generally more abundant in tissues than in biological fluids, it is in the latter that miRNAs quantification seems to be the most interesting. Obtaining a tissue sample requires performing a biopsy, an invasive procedure that can itself cause harmful events for the patient. On the other hand, biological fluids are much more accessible, the sampling is better tolerated by the patient with virtually no risk of adverse effects occurring. Like most biological tests, blood is the fluid of choice for miRNAs analysis. The typical amount of blood required for analysis is 200 μL (*9*).

The analysis can be carried out in whole blood, plasma or serum. Regarding serum and plasma, the results obtained in these two matrices are not similar (*10*, *11*). Thus, McDonald *et al.* found that endogenous miRNAs miR-15b, miR-16 and miR-24 were more expressed in plasma samples than in serum samples (*12*). There is currently no evidence of superiority of one over the other, although plasma appears to be preferable (*13*). Indeed, there is certainly less platelet contamination with serum than with plasma, but the formation of the clot during coagulation can lead to the release of confusing miRNAs from blood cells. In the case of plasma, it is recommended to use ethylenediaminetetraacetatic acid (EDTA) as an anticoagulant. Heparin should be avoided because it could inhibit the polymerase chain reaction (PCR) (*14*). Citrate should also be avoided due to the fact that it could generate hemolysis. Several technologies have been developed to better preserve the RNA species content of whole blood, such as Cell-Free DNA tubes (Streck, Omaha, USA), Cell-Free DNA Collection Tubes (Roche, Basel, Switzerland), PAXgene Blood ccfDNA Tubes (Qiagen, Hombrechtikon, Switzerland) and cf-DNA/cf-RNA Preservative tubes (Norgen Biotek, Thorold, Canada), which can be used for the quantification of miRNAs. These tubes were designed with the purpose of preventing cell lysis and prolonging the storage period of plasma without impacting miRNA analysis. Numerous companies that provide these long-term collection tubes assert that miRNAs can be preserved for a minimum of 7 days in these tubes (*15*). In a direct comparison of these tubes among four different manufacturers, the concentrations of total miRNAs in plasma were not affected when blood was stored at room temperature in PAXgene and Norgen tubes for a duration of up to 1 week. Roche tubes also demonstrated good performance for miRNAs, with only a minor increase in hemolysis observed after 5 and 7 days. On the other hand, Streck tubes exhibited the poorest performance, showing a significant rise in blood cell contamination after 5 days (*16*).

### Centrifugation

Centrifugation conditions have a considerable impact on the results. It is recommended to perform the centrifugation of the samples quickly, within no more than 2 hours after collection. The samples are transported vertically and without agitation in order to prevent hemolysis from occurring. The first centrifugation of the samples should be followed by a second centrifugation or filtration. The aim of the first centrifugation is to eliminate leukocytes and platelets, which are very rich in miRNAs and can constitute a source of errors. Additionally, platelets affect sample preservation, hence the need to remove them before freezing the samples (*17*). The second centrifugation or filtration ensures the elimination of cellular debris and thus obtaining of plasma depleted of platelets suitable for miRNAs determination (*18*). Primary samples should be centrifuged at a low speed, typically ranging from 820 to 3500xg, for a duration of 1-20 minutes at either + 4  °C or room temperature (*10*, *19*, *20*). Following this, a specific volume is carefully pipetted from the supernatant and transferred into a microtube, ensuring that the intermediate layer containing leukocytes and platelets remains undisturbed. Subsequently, a second centrifugation is required, which should be conducted at a speed of 10,000 to 16,000xg for a duration of 15 minutes. The findings of the research conducted by Page *et al.* demonstrate that performing an additional centrifugation at 10,000xg results in a significant reduction in the concentration of platelet-associated miRNAs, including miR-24, miR-191, miR-197 and hsa-miR-223 (*21*).

### Influence of hemolysis

Hemolysis is an important source of interference in the detection of blood miRNAs because it is associated with a significant change in the expression of many miRNAs, particularly those used as endogenous references (*22*). Therefore, is important to minimize *in vitro* hemolysis and blood cell contamination and to document the extent of contamination in each specimen. It should be also noted that hemolyzed samples are poorly preserved by freezing, with a clear difference in the expression of miRNAs before and after freezing. To ensure the absence of hemolysis in the serum or plasma, it is possible to evaluate the miR-451/miR-23 ratio, a high value of this ratio being in favor of hemolysis. Another method consists of measuring the absorbance of hemoglobin spectrophotometrically at 414 nm. An absorbance value greater than 0.2 allows the identification of hemolyzed samples (*23*). The hemolytic index (HI) of serum and plasma samples can be measured using biochemical platforms. The HI values can serve as semiquantitative assessments of the cell-free hemoglobin concentration, with higher values suggesting a more significant hemolysis (*24*).

### Storage

Microribonucleic acids are stable in serum or plasma for up to 24 hours at room temperature or + 4 °C. Long-term storages is possible for at least 1 year at - 20 °C or - 80 °C (*25*). Low temperatures considerably reduce the activity of ribonucleases (RNases), allowing good preservation of samples. The different forms of miRNAs transport protect them from degradation by RNases, making them particularly stable compared to other nucleic acids, such as mRNAs. Striking evidence of this high stability is the presence of intact miRNAs in organs from cryopreserved mummies over 5300 years old (*26*). The results of Grasedieck *et al.* show that frozen samples remain stable for several years and that the miRNA assay on samples stored at - 20 °C gives results similar to those obtained on samples stored at - 80 °C (*27*). This is a major advantage in favor of the use of miRNAs as biomarkers. Indeed, not all laboratories are equipped with - 80 °C freezers, while freezers with temperatures between - 15 °C to - 30 °C are widely available. Freeze/thaw cycles should be avoided.

### Isolation

Conventional methods for isolating total RNA using the guanidinium thiocyanate/phenol/chloroform mixture are not suitable for the isolation of miRNAs. On the one hand, contaminants are often present when using these methods. On the other hand, it has been found that miRNAs with low guanine-cytosine content are selectively lost due to the inefficiency of precipitation of small nucleic acids compared to long nucleic acids (*28*).

Some authors have proposed adapting the phenol-chloroform extraction method by making a few modifications, in particular by removing the washing step with 70% ethanol and allowing the RNA pellet to dry for at least 1 hour at room temperature (*29*). In practice, we instead use commercial kits adapted to miRNAs such as mirVana (Thermo Fischer Scientific, Waltham, USA) or miRNeasy Serum/Plasma (Qiagen, Hombrechtikon, Switzerland). These kits combine separation phase using phenol with purification by adsorption on a silica membrane column. More recent kits like miRNeasy advanced kit (Qiagen, Hombrechtikon, Switzerland) are phenol-free and do not require the phase separation step, which makes it possible to automate the miRNAs isolation process. Due to the low miRNA content in biological fluids, traditional methods like spectrophotometry and fluorimetry should not be used for quantity and quality miRNAs testing. To verify the recovery of a panel of miRNAs of interest quantitative reverse transcription polymerase chain reaction (qRT-PCR) should be used (*30*).

## MicroRNAs expression profiling detection

Northern blotting was the first method used for the detection of miRNAs (*31*). The major disadvantages of this method are its poor sensitivity and the high time consumption. The growing interest in miRNAs has led to the development of detection techniques better suited to these small nucleic acids. Currently, the most used methods for circulating miRNAs expression profiling detection are qRT-PCR, microarrays and next generation sequencing (NGS) (Table 1). Like droplet digital PCR (ddPCR), more recent detection methods have also emerged in recent years. There is no method that can be considered superior to any other. It is crucial to have a comprehensive understanding of the advantages and limits of each method in order to select the most suitable approach based on the research objective.

**Table 1:** Methods for miRNAs expression profiling detection

**Method**	**Advantages**	**Limits**
qRT-PCR	Excellent sensitivity, specificity and accuracy Wide measuring range Suitable for routine measurement in clinical laboratories	Limited to known miRNAs
dd-PCR	Absolute quantification High sensitivity, precision and reproducibility Reduced PCR inhibition effects	Limited to known miRNAs Limited dynamic range Relatively high cost of instrumentation
Microarrays	Profiling of several miRNAs	Limited sensitivity and accuracy
NGS	Allows the discovery of novel miRNAs	High cost Requires extensive bioinformatic analysis
miRNA - microribonucleic acid. qRT-PCR - quantitative reverse transcription polymerase chain reaction. ddPCR - droplet digital polymerase chain reaction. NGS - next generation sequencing.		

### Quantitative reverse transcription polymerase chain reaction

**Figure 1:**
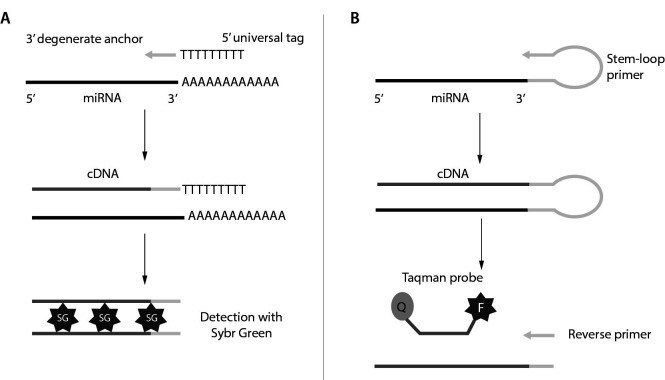
Microribonucleic acids (miRNAs) quantification by quantitative real-time reverse-transcription polymerase chain reaction (qRT-PCR). (A) Poly adenylation method. (B) Stem-loop method. cDNA - complementary DNA.

Thanks to its high sensitivity, its wide measurement range and its high sequence specificity, qRT-PCR constitutes the reference method for measuring miRNAs expression (*32*). Another major advantage of qRT-PCR is its suitability for routine measurement in clinical laboratories, as this equipment is used for a large number of routine applications, such as the search for viruses or mRNAs of clinical interest. The methodology consists, as its name indicates, of a reverse transcription reaction followed by a detection step by quantitative polymerase chain reaction. Due to the low number of nucleotides contained in miRNAs, the reverse transcription step differs somewhat from that of viral RNAs or mRNAs. Two main alternatives are possible to solve the problem of the small size of miRNAs (Figure 1). The first solution, adopted by Qiagen, consists of adding a poly(A) tail to the 3’ end of the miRNA and reverse transcription is carried out using an oligo(dT) primer. To guarantee the specificity of the test, a particular buffer (HiSpec Buffer) is added to the reaction mixture, in order to inhibit the reverse transcription of long coding and non-coding RNAs. The complementary DNA (cDNA) formed is subsequently amplified using two primers, one universal and the other specific for the miRNA to be quantified. The amplification product is measured using the fluorescence emitted by SYBR Green. A fluorescent intercalation that binds to double-stranded nucleic acids with an increase in fluorescence of approximately 800 to 1000 times the baseline signal.

The second approach is based on the use of a primer having a stem-loop (hairpin) type structure. The primer hybridizes to the 3’ end of the miRNA by base complementarity, while the stem-loop part of the primer will stick to the 5’ end of the miRNA. Subsequently, a Taqman probe hybridizes to the cDNA produced. As the DNA polymerase advances, the probe hydrolyzes, which will release the quencher and cause the appearance of a fluorescent light. The choice between these two main approaches to miRNA quantification must be based on the nature of the target. Thus, the strategy used by Qiagen makes it possible to quantify all isomiRs 3’ of the miRNA of interest, while the Taqman method is more specific to a single or a subgroup of isomiRs (*33*).

### Droplet digital polymerase chain reaction

Droplet digital polymerase chain reaction is actually applied to the absolute quantification of miRNAs. The principle of ddPCR consists of compartmentalizing the nucleic acid of interest in a way that only a small quantity of molecules is present in each compartment. The compartments can be in a solid medium in the form of “micro-chambers” or in a liquid medium in the form of emulsions. This limiting dilution means that each compartment contains one or zero target molecules. In the presence of the target molecule, PCR amplification results in the appearance of a fluorescence signal which is read as 1. If conversely, no fluorescent signal is detected, it is interpreted as 0. Data analysis is based on the Poisson probability distribution function, according to total number of compartments, number of fluorescent signals and the dilution coefficient of the sample, the initial value of concentration in the sample can be obtained (*34*). In the study of Binderup *et al.*, the probe-based TaqMan assays were employed to conduct a relative quantification of miRNA in plasma. The correlation between qRT-PCR and ddPCR remained high for miR-126 when normalized to miR-16 or a combination of miR-39 and miR-16, but only moderate for miR-92a (*35*).

Droplet digital polymerase chain reaction has the advantage of being able to overcome the need of a calibration curve and at the same time the influence of standardization strategies. It also has the advantage of being sensitive and presenting a good accuracy for miRNAs measuring of low abundance. The assay is precise and reproducible over a concentration range of four orders of magnitude with sensitivity allowing detection of a target miRNA at concentrations up to 1 copy/μL (*36*). The major disadvantage of this method is the need for specific instruments, reagents and consumables, the cost of which remains significant.

### Microarrays

Microarrays utilize short oligonucleotides that are either deposited or synthesized on a solid support. These oligonucleotides are designed to match the sequence of the miRNAs that are of interest. Through reverse transcription, probes labeled with a fluorochrome or biotin are generated from the miRNAs in solution. These labeled probes are then incubated for a certain period of time and subsequently washed before the signal is measured.

To enhance the analysis process, probes containing locked nucleic acids (LNAs) have been developed. They are derivatives of oligonucleotides in which a methylene group connects the 2’ and 4’ carbon atoms. This modification results in higher melting temperatures (Tm), providing greater thermal stability during molecular hybridization. As a result, the use of LNA probes significantly increases sensitivity and reduces incubation time (*37*).

Microarrays offer the advantage of high throughput and the ability to perform multiplexing, allowing simultaneous analysis of multiple miRNAs. However, their sensitivity is relatively low and the long incubation time hinders routine use. Additionally, a relatively high amount of RNA input (typically 100-2000 ng) is required for analysis. Furthermore, microarrays do not represent a quantitative technique. Therefore, it is crucial to validate the miRNAs of interest using qRT-PCR to ensure accurate and reliable results.

### Next-generation sequencing

Next-generation sequencing (NGS) technologies enable the expression detection of thousands of miRNAs in a single experiment and the discovery of novel miRNAs (*38*). The conventional approach for preparing libraries involves sequential ligations of two defined adapters to the 3’ and 5’ ends of the miRNAs. Subsequently, a universal primer binds to the 3’ adapter to synthesize a cDNA. The resulting cDNA library is then amplified using PCR with primers that are complementary to the adapters. It is worth noting that adapter ligation can also be performed with other species, such as small nucleolar RNA (snoRNA) and PIWI-interacting RNA (piRNA). To remove the ligation products of these species, purification can be achieved through electrophoresis-based size selection (*39*).

A major benefit of NGS is the capacity to detect and measure expression levels of known miRNAs along with those that have yet to be characterized. Another benefit is the ability to concurrently measure expression levels of thousands of miRNAs in one test, including those present at very high or low concentrations in the sample. However, NGS has several drawbacks that restrict its utility. First, it is less accurate than qRT-PCR or microarrays. Second, the high costs, low potential for automation and excessive time consumption for library preparation impede the use of NGS in routine clinical practice. In their study, Godoy *et al.* conducted a comparison of four different platforms, namely RNA-seq, FirePlex, EdgeSeq and nCounter, to analyse the expression of miRNAs associated with the placenta in both pregnant and non-pregnant women. They observed variations in the expression levels of placenta-associated miRNAs in plasma when using RNA-seq and EdgeSeq platforms, but no significant differences detected with FirePlex or nCounter (*40*).

## Normalization of results and interpretation of miRNAs expression profiling detection

**Figure 2:**
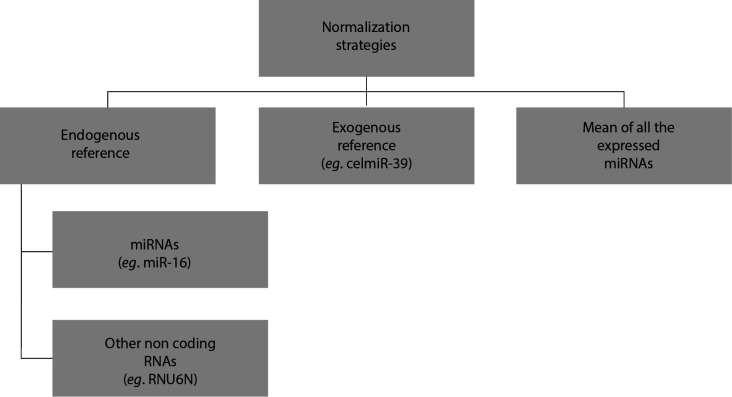
Microribonucleic acids (miRNAs) normalization strategies.

To compensate for the effects of different preanalytical variables such as the quality of isolated miRNAs and the efficiency of reverse transcription, results are expressed in relation to a standard; this is the normalization of results (Figure 2). Due to critical impact of normalization strategy on results interpretation, the choice of normalization reference is of great importance. There are three types of normalization strategies: exogenous reference, endogenous reference or normalization by the average expression value.

### Exogenous reference

To ensure that miRNA quantification is not affected by technical variability that may be introduced during the analysis process, synthetic miRNAs can be used. Among these exogenous non-human miRNAs, we find cel-miR-39 and cel-miR-54. This normalization method is based on the addition before the extraction step of a known quantity of synthetic miRNAs to a defined volume of serum, plasma or whole blood; a stable quantity of exogenous reference is then obtained. Nevertheless, this method is subject of technical and human error. Recently, the scientific community has begun to consider these molecules as unsuitable, due to their reduced apparent stability and because these exogenous molecules are foreign to body fluid samples, and therefore are not perfectly comparable to endogenous miRNAs (*41*).

### Endogenous reference

RNU6B is a snoRNA that is part of the small nuclear ribonucleoprotein U6, a component of the spliceosome responsible for pre-miRNA splicing. The use of RNU6B and other snoRNAs in miRNA-related research is controversial. These larger molecules are likely to be less reliable than miRNAs because their expression is less stable than miRNAs with studies showing more frequent degradation in serum samples. It is therefore difficult to draw conclusions regarding miRNAs expression when snoRNAs are used as endogenous reference. A large number of studies have employed RNU6B as an endogenous reference for miRNA quantification (*42*-*44*). However, RNU6B should not be used for normalization strategy. On the one hand, blood concentrations of RNU6B show high interindividual variability, up to eight cycles in qRT-PCR (*45*). On the other hand, the determination of the expression of RNU6B and miR-16 in serum subjected to different freezing/thawing cycles revealed that the expression of RNU6B gradually decreases after 1, 2 and 4 freezing/thawing cycles, while the expression of miR-16 remains relatively stable (*46*).

MiR-16 is one of the most commonly used miRNAs as an endogenous reference in the literature (*41*). Kloten *et al.* conducted a study comparing different extraction methods of circulating miRNAs and protocols for isolating miRNAs in exosomes (*47*). They evaluated the effectiveness of these extraction methods by measuring an exogenous control, cel-miR-39, and 6 endogenous miRNAs in plasma. Among these miRNAs, miR-16 was found to be the most efficient. It was not only abundantly represented compared to the other tested miRNAs, but also its use as an endogenous control for normalizing threshold cycle values significantly reduced variability between different extraction methods. However, the use of miR-16 as an endogenous control is controversial in certain pathological conditions, such as breast cancer. Holubekova *et al.* conducted a study involving plasma samples from breast cancer patients and healthy female volunteers (*48*). They screened selected miRNAs using qRT-PCR in pilot tests and identified miR-16 as the most stable reference gene for breast cancer normalization using the geNorm algorithm. McDermott *et al.* also analysed the expression of numerous miRNAs in blood samples from breast cancer patients and controls (*49*). The geNorm algorithm determined that miR-425 and miR-16 were the most stable reference miRNAs among the candidates. The expression of miR-425 and miR-16 was further confirmed in a larger cohort of breast cancer patients and healthy women, which validated their stable expression in both groups. In contrast, Stückrath *et al.* reported significantly increased plasma concentrations of miR-16 in patients without lymph node involvement, while patients with lymph node metastases exhibited decreased or even normal values (*50*). In their study, Shin *et al.* discovered a significant downregulation of miR-16 in plasma samples obtained from patients with triple-negative breast cancer when compared to healthy individuals (P < 0.001) (*51*). Based on their findings, the researchers suggested that miR-16 could serve as a potential biomarker for the diagnosis of triple-negative breast cancer. MiR-484 was utilized as an endogenous reference in this study.

## Potential future developments

**Figure 3:**
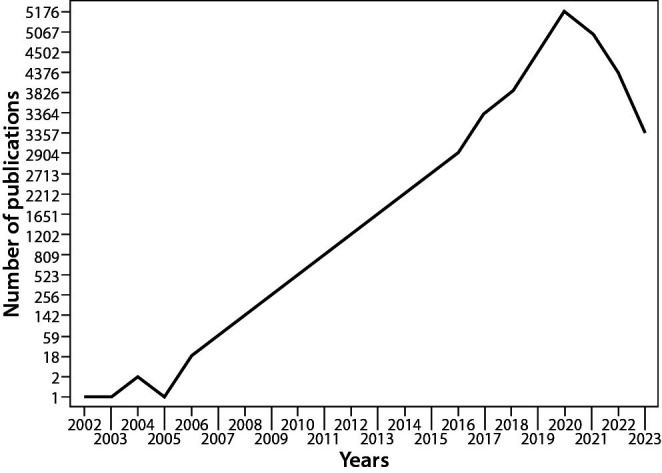
Number of publications *per* year on PubMed by typing the following keywords: “microRNAs” and “biomarkers”.

The number of research studies assessing the advantages of circulating miRNAs has grown significantly in the past few years (Figure 3). However, currently no miRNAs signature is applied in the clinical setting. The primary cause of the disparities observed in research studies and the inability of the results to be applied to clinical practice thus far can be attributed to the lack of consistency in the methods used to quantify circulating miRNAs. The National Cancer Institute (NCI) has recently developed a new Biospecimen Evidenced-Based Practices (BEBP) paper titled “Cell-free miRNA (cfmiRNA): Blood Collection and Processing” in order to meet the need for standardization and evidence-based recommendations (*52*). The fourth document in the series, the BEBP, provides detailed procedural instructions designed especially for cfmiRNAs analysis of plasma and serum. These guidelines cover blood collection, processing, storage, extraction and quality assessment. Although this text is a significant improvement, much work remains. In fact, the preanalytical phase is the primary focus of the recommendations made in this document. Expert consensus on miRNAs assays and results normalization approaches are necessary. In addition, it is imperative to establish distinct analysis protocols for alternative types of circulating miRNAs that do not exist in a free state, such as exosomal miRNAs.

## Conclusion

To obtain reproducible and informative results for miRNAs, it is crucial to strictly standardize the entire process, starting from collecting samples to reporting findings. Regardless of the methodology utilized in a research study on miRNAs, it is essential to rigorously apply it to all samples from patients and controls alike. This ensures that the expression differences seen are genuinely due to the pathology under study, rather than variability introduced by the analytical process. The standardization of miRNAs measurement in biofluids is of great importance in the field of research to make the results comparable from one study to another and lead to the identification of circulating miRNAs with concrete clinical applications. This will also lead to a better understanding of the involvement of miRNAs in the physiopathology of human diseases and the development of new therapeutic agents.

## Data Availability

No data was generated during this study, so data sharing statement is not applicable to this article.

## References

[1] Du T Zamore PD. microPrimer: the biogenesis and function of microRNA. Development. 2005;132:4645–52. 10.1242/dev.02070 16224044

[2] Mitchell PS Parkin RK Kroh EM Fritz BR Wyman SK Pogosova-Agadjanyan EL Circulating microRNAs as stable blood-based markers for cancer detection. Proc Natl Acad Sci USA. 2008;105:10513–8. 10.1073/pnas.0804549105 18663219 PMC2492472

[3] Sohel MMH. Circulating microRNAs as biomarkers in cancer diagnosis. Life Sci. 2020;248:117473. 10.1016/j.lfs.2020.117473 32114007

[4] Lawrie CH Gal S Dunlop HM Pushkaran B Liggins AP Pulford K Detection of elevated levels of tumour-associated microRNAs in serum of patients with diffuse large B-cell lymphoma. Br J Haematol. 2008;141:672–5. 10.1111/j.1365-2141.2008.07077.x 18318758

[5] Zendjabil M. Circulating microRNAs as novel biomarkers of Alzheimer’s disease. Clin Chim Acta. 2018;484:99–104. 10.1016/j.cca.2018.05.039 29800558

[6] Lippi G Mattiuzzi C Bovo C. Are we getting better at the preanalytical phase or just better at measuring it? J Lab Precis Med. 2018;3:11. 10.21037/jlpm.2018.01.03

[7] Heegaard NHH Carlsen AL Lilje B Ng KL Rønne ME Jørgensen HL Diurnal Variations of Human Circulating Cell-Free Micro-RNA. PLoS One. 2016;11:e0160577. 10.1371/journal.pone.0160577 27494182 PMC4975411

[8] Pritchard CC Cheng HH Tewari M. MicroRNA profiling: approaches and considerations. Nat Rev Genet. 2012;13:358–69. 10.1038/nrg3198 22510765 PMC4517822

[9] Sourvinou IS Markou A Lianidou ES. Quantification of circulating miRNAs in plasma: effect of preanalytical and analytical parameters on their isolation and stability. J Mol Diagn JMD. 2013;15:827–34. 10.1016/j.jmoldx.2013.07.005 23988620

[10] Foye C Yan IK David W Shukla N Habboush Y Chase L Comparison of miRNA quantitation by Nanostring in serum and plasma samples. PLoS One. 2017;12:e0189165. 10.1371/journal.pone.0189165 29211799 PMC5718466

[11] Mompeón A Ortega-Paz L Vidal-Gómez X Costa TJ Pérez-Cremades D Garcia-Blas S Disparate miRNA expression in serum and plasma of patients with acute myocardial infarction: a systematic and paired comparative analysis. Sci Rep. 2020;10:5373. 10.1038/s41598-020-61507-z 32214121 PMC7096393

[12] McDonald JS Milosevic D Reddi HV Grebe SK Algeciras-Schimnich A. Analysis of circulating microRNA: preanalytical and analytical challenges. Clin Chem. 2011;57:833–40. 10.1373/clinchem.2010.157198 21487102

[13] Dufourd T Robil N Mallet D Carcenac C Boulet S Brishoual S Plasma or serum? A qualitative study on rodents and humans using high-throughput microRNA sequencing for circulating biomarkers. Biol Methods Protoc. 2019;4:bpz006. 10.1093/biomethods/bpz006 32395624 PMC7200924

[14] Glinge C Clauss S Boddum K Jabbari R Jabbari J Risgaard B Stability of Circulating Blood-Based MicroRNAs - Pre-Analytic Methodological Considerations. PLoS One. 2017;12:e0167969. 10.1371/journal.pone.0167969 28151938 PMC5289450

[15] Markou AN Lianidou ES. The Impact of Pre-analytical Factors on the Reliability of miRNA Measurements. Curr Pathobiol Rep. 2019;7:29–33. 10.1007/s40139-019-00191-9

[16] Ward Gahlawat A Lenhardt J Witte T Keitel D Kaufhold A Maass KK Evaluation of Storage Tubes for Combined Analysis of Circulating Nucleic Acids in Liquid Biopsies. Int J Mol Sci. 2019;20:704. 10.3390/ijms20030704 30736351 PMC6387045

[17] Mitchell AJ Gray WD Hayek SS Ko YA Thomas S Rooney K Platelets confound the measurement of extracellular miRNA in archived plasma. Sci Rep. 2016;6:32651. 10.1038/srep32651 27623086 PMC5020735

[18] Binderup HG Houlind K Madsen JS Brasen CL. Pre-storage centrifugation conditions have significant impact on measured microRNA levels in biobanked EDTA plasma samples. Biochem Biophys Rep. 2016;7:195–200. 10.1016/j.bbrep.2016.06.005 28955906 PMC5613297

[19] Murray MJ Watson HL Ward D Bailey S Ferraresso M Nicholson JC “Future-Proofing” Blood Processing for Measurement of Circulating miRNAs in Samples from Biobanks and Prospective Clinical Trials. Cancer Epidemiol Biomarkers Prev. 2018;27:208–18. 10.1158/1055-9965.EPI-17-0657 29254935 PMC5812437

[20] Tanriverdi K Kucukural A Mikhalev E Tanriverdi SE Lee R Ambros VR Comparison of RNA isolation and associated methods for extracellular RNA detection by high-throughput quantitative polymerase chain reaction. Anal Biochem. 2016;501:66–74. 10.1016/j.ab.2016.02.019 26969789

[21] Page K Guttery DS Zahra N Primrose L Elshaw SR Pringle JH Influence of plasma processing on recovery and analysis of circulating nucleic acids. PLoS One. 2013;8:e77963. 10.1371/journal.pone.0077963 24205045 PMC3799744

[22] Myklebust MP Rosenlund B Gjengstø P Bercea BS Karlsdottir Á Brydøy M Quantitative PCR Measurement of miR-371a-3p and miR-372-p Is Influenced by Hemolysis. Front Genet. 2019;10:463. 10.3389/fgene.2019.00463 31191602 PMC6539204

[23] Kirschner MB Kao SC Edelman JJ Armstrong NJ Vallely MP van Zandwijk N Haemolysis during sample preparation alters microRNA content of plasma. PLoS One. 2011;6:e24145. 10.1371/journal.pone.0024145 21909417 PMC3164711

[24] Poel D Buffart TE Oosterling-Jansen J Verheul HM Voortman J. Evaluation of several methodological challenges in circulating miRNA qPCR studies in patients with head and neck cancer. Exp Mol Med. 2018;50:e454. 10.1038/emm.2017.288 29520111 PMC5898892

[25] Khan J Lieberman JA Lockwood CM. Variability in, variability out: best practice recommendations to standardize pre-analytical variables in the detection of circulating and tissue microRNAs. Clin Chem Lab Med. 2017;55:608–21. 10.1515/cclm-2016-0471 28306519

[26] Keller A Kreis S Leidinger P Maixner F Ludwig N Backes C miRNAs in Ancient Tissue Specimens of the Tyrolean Iceman. Mol Biol Evol. 2017;34:793–801. 10.1093/molbev/msw291 28025275

[27] Grasedieck S Schöler N Bommer M Niess JH Tumani H Rouhi A Impact of serum storage conditions on microRNA stability. Leukemia. 2012;26:2414–6. 10.1038/leu.2012.106 22504138

[28] Kim YK Yeo J Kim B Ha M Kim VN. Short structured RNAs with low GC content are selectively lost during extraction from a small number of cells. Mol Cell. 2012;46:893–5. 10.1016/j.molcel.2012.05.036 22749402

[29] Choi C Yoon S Moon H Bae YU Kim CB Diskul-Na-Ayudthaya P mirRICH, a simple method to enrich the small RNA fraction from over-dried RNA pellets. RNA Biol. 2018;15:763–72. 10.1080/15476286.2018.1451723 29638187 PMC6152462

[30] Ford KL Anwar M Heys R Ahmed EM Caputo M Game L Optimisation of laboratory methods for whole transcriptomic RNA analyses in human left ventricular biopsies and blood samples of clinical relevance. PLoS One. 2019;14:e0213685. 10.1371/journal.pone.0213685 30870483 PMC6417664

[31] Várallyay E Burgyán J Havelda Z. MicroRNA detection by northern blotting using locked nucleic acid probes. Nat Protoc. 2008;3:190–6. 10.1038/nprot.2007.528 18274520

[32] Kappel A Keller A. miRNA assays in the clinical laboratory: workflow, detection technologies and automation aspects. Clin Chem Lab Med. 2017;55:636–47. 10.1515/cclm-2016-0467 27987355

[33] Dellett M Simpson DA. Considerations for optimization of microRNA PCR assays for molecular diagnosis. Expert Rev Mol Diagn. 2016;16:407–14. 10.1586/14737159.2016.1152184 26854938

[34] Denis JA Nectoux J Lamy PJ Rouillac Le Sciellour C Guermouche H Alary AS Development of digital PCR molecular tests for clinical practice: principles, practical implementation and recommendations. Ann Biol Clin (Paris). 2018;76:505–23. 30226193 10.1684/abc.2018.1372

[35] Binderup HG Madsen JS Heegaard NHH Houlind K Andersen RF Brasen CL. Quantification of microRNA levels in plasma - Impact of preanalytical and analytical conditions. PLoS One. 2018;13:e0201069. 10.1371/journal.pone.0201069 30024941 PMC6053236

[36] Ferracin M Negrini M. Quantification of Circulating MicroRNAs by Droplet Digital PCR. Methods Mol Biol. 2018;1768:445–57. 10.1007/978-1-4939-7778-9_25 29717458

[37] Ye J Xu M Tian X Cai S Zeng S. Research advances in the detection of miRNA. J Pharm Anal. 2019;9:217–26. 10.1016/j.jpha.2019.05.004 31452959 PMC6702429

[38] Liu P. MicroRNA Expression Analysis: Next-Generation Sequencing. Methods Mol Biol. 2018;1783:171–83. 10.1007/978-1-4939-7834-2_8 29767362

[39] Buschmann D Haberberger A Kirchner B Spornraft M Riedmaier I Schelling G Toward reliable biomarker signatures in the age of liquid biopsies - how to standardize the small RNA-Seq workflow. Nucleic Acids Res. 2016;44:5995–6018. 10.1093/nar/gkw545 27317696 PMC5291277

[40] Godoy PM Barczak AJ DeHoff P Srinivasan S Etheridge A Galas D Comparison of Reproducibility, Accuracy, Sensitivity, and Specificity of miRNA Quantification Platforms. Cell Rep. 2019;29:4212–22.e5. 10.1016/j.celrep.2019.11.078 31851944 PMC7499898

[41] Donati S Ciuffi S Brandi ML. Human Circulating miRNAs Real-time qRT-PCR-based Analysis: An Overview of Endogenous Reference Genes Used for Data Normalization. Int J Mol Sci. 2019;20:E4353. 10.3390/ijms20184353 31491899 PMC6769746

[42] Swellam M El Magdoub HM Hassan NM Hefny MM Sobeih ME. Potential diagnostic role of circulating MiRNAs in breast cancer: Implications on clinicopathological characters. Clin Biochem. 2018;56:47–54. 10.1016/j.clinbiochem.2018.04.013 29679553

[43] El-Shafae M Behiry EG. Abd El-latif ME, Ahmed ES, El-Fallah AA. Clinical value of serum microRNA-195 expression in invasive ductal carcinoma of the breast. Gene Rep. 2020;19:100635. 10.1016/j.genrep.2020.100635

[44] Saleh AA Soliman SE Habib MSED Gohar SF Abo-Zeid GS. Potential value of circulatory microRNA122 gene expression as a prognostic and metastatic prediction marker for breast cancer. Mol Biol Rep. 2019;46:2809–18. 10.1007/s11033-019-04727-5 30835039

[45] Benz F Roderburg C Vargas Cardenas D Vucur M Gautheron J Koch A U6 is unsuitable for normalization of serum miRNA levels in patients with sepsis or liver fibrosis. Exp Mol Med. 2013;45:e42. 10.1038/emm.2013.81 24052167 PMC3789266

[46] Xiang M Zeng Y Yang R Xu H Chen Z Zhong J U6 is not a suitable endogenous control for the quantification of circulating microRNAs. Biochem Biophys Res Commun. 2014;454:210–4. 10.1016/j.bbrc.2014.10.064 25450382

[47] Kloten V Neumann MHD Di Pasquale F Sprenger-Haussels M Shaffer JM Schlumpberger M Multicenter Evaluation of Circulating Plasma MicroRNA Extraction Technologies for the Development of Clinically Feasible Reverse Transcription Quantitative PCR and Next-Generation Sequencing Analytical Work Flows. Clin Chem. 2019;65:1132–40. 10.1373/clinchem.2019.303271 31235535

[48] Holubekova V Kolkova Z Grendar M Brany D Dvorska D Stastny I Pathway Analysis of Selected Circulating miRNAs in Plasma of Breast Cancer Patients: A Preliminary Study. Int J Mol Sci. 2020;21:E7288. 10.3390/ijms21197288 33023154 PMC7583045

[49] McDermott AM Kerin MJ Miller N. Identification and validation of miRNAs as endogenous controls for RQ-PCR in blood specimens for breast cancer studies. PLoS One. 2013;8:e83718. 10.1371/journal.pone.0083718 24391813 PMC3877087

[50] Stückrath I Rack B Janni W Jäger B Pantel K Schwarzenbach H. Aberrant plasma levels of circulating miR-16, miR-107, miR-130a and miR-146a are associated with lymph node metastasis and receptor status of breast cancer patients. Oncotarget. 2015;6:13387–401. 10.18632/oncotarget.3874 26033453 PMC4537022

[51] Shin VY Siu JM Cheuk I Ng EKO Kwong A. Circulating cell-free miRNAs as biomarker for triple-negative breast cancer. Br J Cancer. 2015;112:1751–9. 10.1038/bjc.2015.143 25906045 PMC4647231

[52] Greytak SR Engel KB Hoon DSB Elias KM Lockwood CM Guan P Evidence-based procedures to improve the reliability of circulating miRNA biomarker assays. Clin Chem Lab Med. 2023;62:60–6. 10.1515/cclm-2023-0131 37129007

